# The health Oriented pedagogical project (HOPP) - a controlled longitudinal school-based physical activity intervention program

**DOI:** 10.1186/s12889-017-4282-z

**Published:** 2017-04-28

**Authors:** Per Morten Fredriksen, Ole Petter Hjelle, Asgeir Mamen, Trine J. Meza, Ane C. Westerberg

**Affiliations:** Kristiania University College - Department of Health Sciences, PB 1195 Sentrum, 0107 Oslo, Oslo Norway

**Keywords:** Cardio-metabolic, Risk factors, School, Physical activity, Program, Children

## Abstract

**Background:**

The prevalence of non-communicable diseases (NCDs) is increasing worldwide, also among children. Information about primary prevention of NCD’s is increasing; however, convincing strategies among children is needed. The present paper describes the design and methods in the Health Oriented Pedagogical Project (HOPP) study. The main objective is to evaluate the effects of a school-based physical activity intervention program on cardio-metabolic risk factors. Secondary objectives include assessment of physical, psychological and academic performance variables.

**Methods:**

The HOPP study is a 7 years longitudinal large-scale controlled intervention in seven elementary schools (*n* = 1545) with two control schools (*n* = 752); all aged 6–11 years at baseline. The school-based physical activity intervention program includes an increase in physical activity (PA) of 225 min/week as an integrated part of theoretical learning, in addition to the curriculum based 90 min/week of ordinary PA. Primary outcomes include cardio-metabolic risk factors measured as PA level, BMI status, waist circumference, muscle mass, percent fat, endurance test performance, total serum cholesterol, high-density lipoprotein (HDL), non-HDL, micro C-reactive protein (mCRP) and long-term blood sugar (HbA1c). In addition, secondary outcomes include anthropometric growth measures, physical fitness, quality of life (QoL), mental health, executive functions, diet and academic performance.

**Discussion:**

HOPP will provide evidence of effects on cardio-metabolic risk factors after a long-term PA intervention program in elementary schoolchildren. School-based PA intervention programs may be an effective arena for health promotion and disease prevention.

**Trial registration:**

The study is registered in Clinical trials (ClinicalTrials.gov Identifier: NCT02495714) as of June 20^th^ – 2015, retrospectively registered. The collection of baseline values was initiated in mid-January 2015.

## Background

Cardio-metabolic risk factors in childhood may act both as a measure of children’s health status, as well as a risk prediction of later illness. Several international studies have revealed increasing prevalence of adiposity in children and adolescents, and 41 million children younger than 5 years are estimated to be overweight or obese [1, 2]. In Norway, secular trends have shown an increase in adiposity, and a call for large scale monitoring of weight in children and adolescents has been proposed [3–5]. Hence, a significant proportion of children are at risk of developing lifestyle diseases as well as related emotional and psychosocial problems [1]. Lifestyle diseases earlier only seen in adults are wide spreading amongst adolescents, and even children [1]. In an attempt to counteract the immense lifestyle related problems worldwide preventing strategies have emerged [6].

There are few arenas where all layers of the population may be reached when implementing preventive measures to reduce sedentary lifestyle. To enhance physical activity (PA) and promote healthy behaviour, elementary schools are suitable arenas to reach all children [7]. Several studies have combined PA interventions with a pedagogic approach in elementary schools around the world [7–10] These studies have indicated a positive effect in terms of reduced overweight and associated diseases, as well as improved cognitive function and mental health [4, 11–18].

Most children spend a large part of their daily life at school, and schools offer a unique opportunity to promote health and wellbeing among *all* children with different socioeconomic background. The use of a school-based PA program may be an important tool to provide health promotion and disease prevention. Based on previous research a well-designed school-based PA intervention trial with a longitudinal design measuring health-related factors with a sufficient number of participants over a long time-period is overdue.

**Fig. 1:**
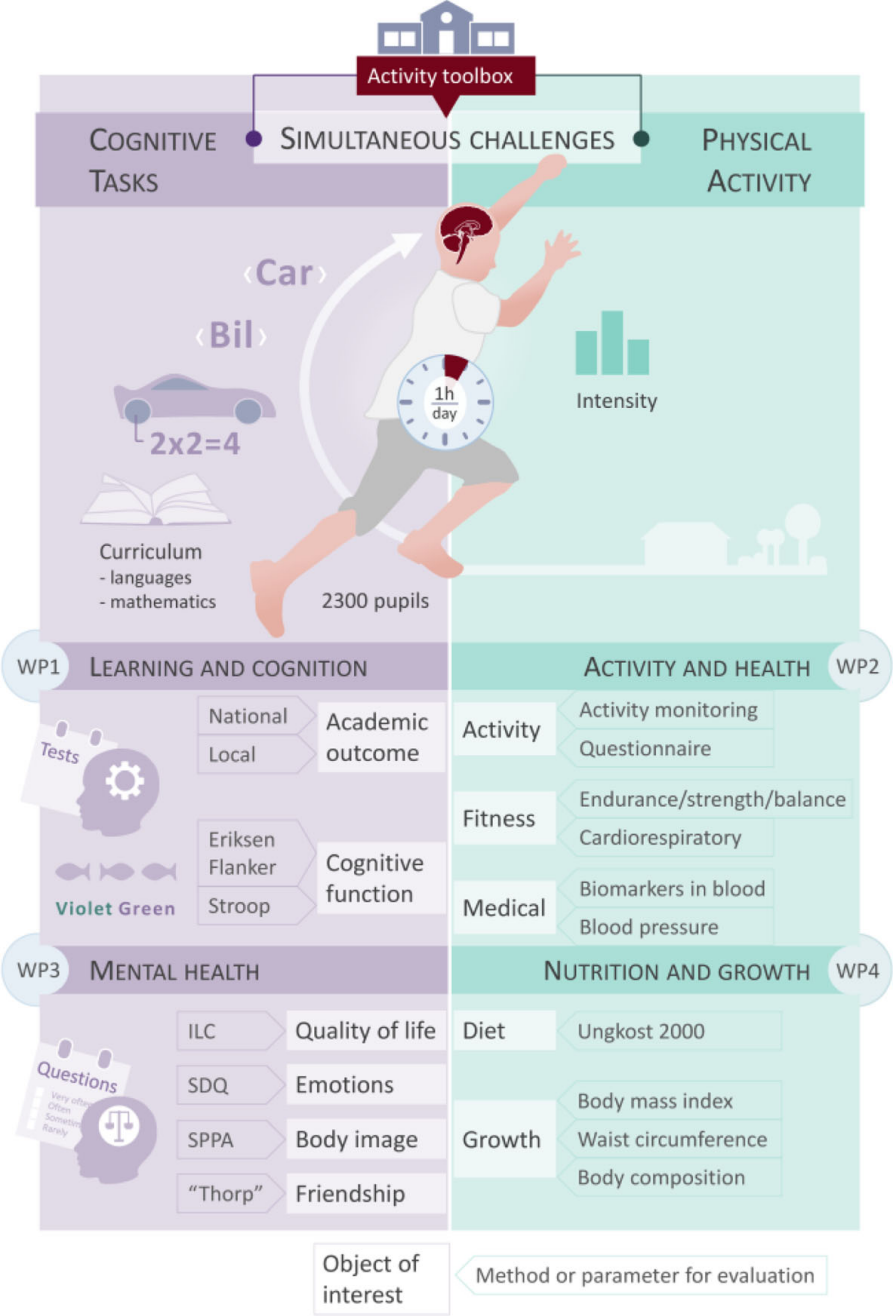
Overview of the thematic areas in the HOPP-project

In 2014, politicians in Horten municipality, Norway, initiated a health promotion program for children called the Health Oriented Pedagogical Project (HOPP). HOPP was initiated in response to an increasing prevalence of obesity, inactivity and lifestyle related diseases in the local child population. The main objective is to evaluate the effects of a school-based PA intervention program on cardio-metabolic risk, offering a proxy for later non-communicable disease. Secondary objectives include assessment of physical, psychological and academic performance variables (Fig. 1).

## Methods/design

### Study population and inclusion criteria

The HOPP study is a controlled longitudinal school-based PA intervention. Seven elementary schools in Horten municipality, Vestfold County, Norway are intervention schools. Two control schools in Akershus County, Eiksmarka school (*n* = 520) and Rasta school (*n* = 465), were included. The control schools were recruited based on estimated socioeconomic level, using a centralised Norwegian program for systematic quality work in schools and kindergartens called PULS. This represent a total population of 2817 pupils of which 1815 children received the intervention as a part of the pedagogical platform in Horten municipality. All children were invited to be part of the research project. Based on recruitment procedures a total baseline participation of 2297 pupils (82%) was achieved. The children were recruited from 1^st^ - 6^th^ grade (age 6–11 yrs) (Table 1).

**Table 1:** Baseline population for all grades as of 2015 is shown, as well as the sample size, based on children whose parents gave consent to participation in the research project

Grades	1	2	3	4	5	6	Total
Population	457	484	457	462	461	496	2817
Sample	351	381	370	389	381	425	2297
Intervention schools	248	264	229	264	269	271	1545
Control schools	103	117	141	125	112	154	752

The sample size is divided into intervention and control schools

### Information and recruitment

The school-based PA program was compulsory and implemented as a new pedagogical standard within the Horten municipality. An information campaign about HOPP was performed by Horten municipality in 2014, including written information with informed consent to parents, pamphlets, social media interaction and a website. In addition, the study team gave oral and written information to parents at school meetings. No information about the school-based PA program was given to control schools.

### Intervention

The main aim in HOPP is to reduce cardio-metabolic risk factors in elementary school children by increasing PA in school as part of the pedagogical approach. As elementary school is compulsory, children from all socioeconomic layers will be reached. The implementation is low cost as both physical (school facilities) and human (teachers) resources are already available.

A working group of 14 experienced teachers (two from each school) in Horten municipality with special interest and education in health promotion and PA, as well as pedagogical experience were assembled. Harter’s Competence Motivation Theory was the theoretical basis used by the working group [19, 20]. The theory is achievement motivation based, and founded on a person’s feelings of personal competence. According to the social learning theory of Bandura, self-efficacy increases when a person successfully masters a task. This encourages the person to master more tasks. Embracing this theory, our school-based PA program is based on a constant development of mastery of the curriculum throughout elementary school [19, 20]. In HOPP, the educational program is re-structured from passive learning into active learning. Thus, using the natural potential in the child for PA to enforce academic skills and simultaneously promoting health.

The working group established a large library of activities emphasizing theoretical learning while being physically active, within language, mathematics and English subjects within academic guidelines for grades 1–7. The activities were based both on the teachers’ own ideas and proposals from The Norwegian Directorate for Education and Training (udir.no) and ideas from Trudvang Elementary School. The activities were adjusted for grade level and aimed to have moderate to high intensity level, with 25–30% of the time at a vigorous activity level.

“Activity boxes” according to the grade level were created following an eight months testing period. All boxes, located in each classroom, contained teaching material with the possibility of progression. After the eight months testing period, all 210 teachers in Horten municipality were trained in the new pedagogical approach enabling the use of PA in school lessons. This was done in spring 2015 by: ^1)^ a two-day gathering of all teachers, ^2)^ two dedicated teachers from the working group held follow-up courses at each school with regular intervals in spring 2015 and ^3)^ the municipality established a 100% position for a specially trained teacher to follow up all schools weekly during the whole project period (2015–2021).

In Norway, organised PA during elementary school consists of 90 min weekly physical education lessons. The HOPP intervention in Horten municipality consisted of additional 45 min of activity a day, replacing ordinary desk learning with physical tasks. Hence, the school-based PA program at the intervention schools involves an addition of 225 min/week of PA. Based on the “library” of activities in the toolbox, teachers decided individually when and how the activity lesson should be conducted. As a morning session in elementary school typically lasts 90 min before recess, a typical lesson often consists of 45 min of theory in classroom, then 45 min with active learning. The activities were performed in the schoolyard, gymnasium or in the school halls.

No individual follow-up of pupil’s participation in the intervention was completed. This implies that individual intensity level and absence from school are not recorded. However, teachers completed daily a questionnaire regarding the number of minutes of activity and intensity level of each class. Twice a year the questionnaires were collected. The control schools do not have any PA program or plans for establishing any program during the project period. The control schools upheld their 90 min/week according to the curriculum.

### Outcome measures

The tests were supervised by the principal investigator, and conducted by students from the Kristiania University College, Institute of Health Sciences, Oslo, Norway. All students were trained before administrating the tests. All tests were performed at the elementary schools.

**Fig. 2:**
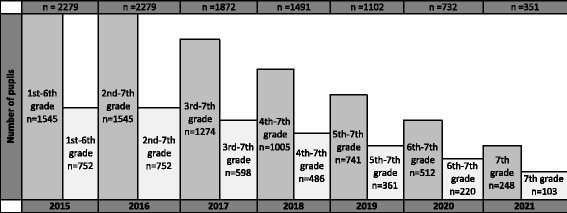
Estimated number of pupils tested annually, assuming no dropouts. Dark grey is intervention schools, light grey control schools

**Fig. 3:**
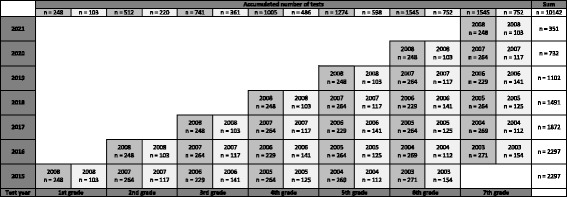
The accumulated number of annual tests at each grade is shown. Each cohort’s birth year is located within each column along with the estimated number of participants, which equals sample size. Dark grey columns are intervention schools, light grey control schools

All pupils in 1st to 6th grade (6–11 years) recruited in 2015 are followed longitudinally from baseline in spring 2015 for as long as they stay in elementary school. Hence, 1st grades in 2015 are followed until 2021, though 6th grade is followed for one year. This implies an annually decline in the number of participants in the research project as the eldest pupils moves on to junior high school (Fig. 2). The implementation of the intervention started fall 2015. Due to ^1)^ time limitations, ^2)^ the total test load at each grade and ^3)^ age specific tests, some tests were limited to certain age groups, as shown in Table 2. In Fig. 3 the cumulative number of all tests at each grade throughout the length of the study is shown.

**Table 2:** Test variables for HOPP

Annual test methods	Grades
Activity & health	
Physical activity monitoring	All
Fitness tests^b^	All
Oxygen uptake^c^	1^st^
Blood samples^d^	All who volunteered
Learning & cognition	
Erikson Flanker test	2^nd^
Stroop test	4^th^, 5^th^ & 6^th^
Academic national tests	5^th^
Nutrition and growth	
Diet (“Ungkost 2000”)	4th
Anthropometry	All
Body composition^a^	All
Mental health	
Life Quality in Children and Adolescents (ILC)	All
Strengths and Difficulties Questionnaire (SDQ) (2016)^e^	6^th^ & 7^th^
Self-Perception Profile for Adolescents (SPPA) (2016)^e^	6^th^ & 7^th^

^a^= Using Tanita bio-impedance measurements.

^b^= Fitness tests include endurance test (Andersen test), strength (handgrip) and balance.

^c^= Oxygen uptake was performed on 1st grade pupils only as it is very time consuming.

^d^= Blood samples include serum cholesterol, High-density Lipoprotein (HDL), Ferritin, Iron, Cobalamin (total vitamin B12), Holotranscobalamin (active vitamin B12), Creatinine, HS-CRP, blood count, Haemoglobin, Hematocrit and HbA1c.

^e^= SDQ & SPPA are tested as of 2016.

### Children’s test

#### Annual measurements for all participants

Measurement of ^1)^ anthropometry, ^2)^ body composition, ^3)^ fitness and ^4)^ PA level, ^5)^ blood pressure, ^6)^ blood samples and ^7)^ The Inventory of Life Quality in children and adolescents (ILC) are to be completed annually for all participants (Table 3). The protocol design allows for baseline analyses, interventional one-year effect analyses, analyses of secular trends and longitudinal effect analyses across the full length of the study. Due to being very time consuming oxygen uptake is restricted to pupils born 2008 (1^st^ grade in 2015).

**Table 3:** Based on the various power tests, the following sample size (intervention and controls schools) was calculated, with a beta of 80% and alpha of 5%

Test area	n
Anthropometry	64^b^
Cardio-metabolic risk factors	126^c^
Physical activity level	73^a^
Aerobic capacity	10^a^
General physical capacity	126^c^
Blood tests	34^a^
Quality of life	73^a^
Diet	24^a^
Cognitive ability	85^a^
Academic performance	34^a^

Statistical program: Systat 13, Power Analysis. ^a^One-sample t-test, ^b^Two-sample t-test, ^c^Z-score

##### 1) Anthropometric measures

Waist circumference was measured to the nearest 0.5 cm with an anthropometric tape, at full expiration at the level of the umbilicus. Body height was measured to the nearest half cm, without shoes, using (SECA GmbH, Germany). Body mass was measured barefooted, in light clothing, using an electronic scale (Tanita MC-980MA, Tokyo, Japan). To compensate for the weight of clothes 0,4 kg was withdrawn from the total weight. Based on weight and height the isoBMI was calculated.

##### 2) Body composition

A bio-impedance Tanita scale (Tanita MC-980MA, Tokyo, Japan) was used to estimate muscle mass (kg), body fat (kg and %), bone mass (kg) and the amount of protein (kg).

##### 3) Fitness

Using Jamar handgrip (Jamar Dynamometer, Lafayette, USA) the pupils were instructed to stand firmly on both feet, with a straight elbow keeping the right arm tucked to the body. They were then told to squeeze as hard as possible for 2–3 s. After reading the results, the procedure was repeated. If the pupil misunderstood the instructions or clearly underachieved, a third test was executed. The highest value was recorded. An international reference material are to be used for comparison of results [21].

Balance Y-test (FM-Systems, USA) is based on a set of tests with repeated measurements [22, 23]. The modified test started with the right foot placed on the platform, toes touching the red mark. Starting with a forward push, then a left push and a last push with the left leg crossed behind the right leg. A test was considered approved when the pupil managed to push the block with the toe without leaning on the block or losing the balance after a full stretch. All pupils were instructed and were allowed three trials. Failure to complete a test was marked. The result was measured in centimetres. When analysing the results, the score was divided by age and height of the pupil.

Aerobic fitness was measured using the Andersen intermittent running test in all pupils [24]. Gymnasiums in the attending schools are 20 m wall-to-wall, covered with wooden or rubber floor. The pupils had to touch the wall with one hand, turn around and run back. Music signalled 15 s of running and 15 s of rest during 10 min, giving an overall running time of five minutes. Supervisors controlled the running distance by checking on a form the number of times the pupils run back and forth. After the last lap the length in meters were registered. Subjects were told to run as fast as they could in order to cover the longest possible distance [24].

##### 4) Objective assessment of PA level

An activity monitor (Actigraph wGT3X-BT, ActiGraph LLC, Pensacola, FL, USA) was attached on the hip with an elastic band and worn for 7 consecutive days and nights unless injured, ill or absent. Assessment of activity level will be assessed annually in the same time period to try to ensure the same weather conditions as during baseline testing.

The sampling frequency used was 100 Hz and 10 s epoch. To be included in the analysis, 480 min of registered activity per day was acquired. The minimum of active days was four, and sleep was defined between 22:00–06:00. The intensity domains of sedentary (0–99 counts), light (100–1999), moderate (2000–4999) and vigorous (5000 - _∞_) were used as according to the latest update from ActiGraph LLC, Pensacola, FL, USA. Outcomes for PA levels were mean counts per minute during all assessed minutes as well as time (minutes) spent in different intensity domains (sedentary, light, moderate, vigorous PA). These parameters were investigated in segments of the day (school time, recess, and leisure-time) as class-specific time tables were collected at both assessment periods. These segments were all components in the HOPP intervention and makes it possible to investigate whether the intervention caused any changes in PA during the different segments of the day [8, 18].

##### 5) Blood pressure

Blood pressure is measured automatically (Model M3 Intellisense HEM-7051-E, Omron Healthcare, Kyoto, Japan) on the upper left arm after being seated on a chair for a few minutes. When fail to detect pressure was apparent, a new test was done immediately. After three unsuccessful tests the cuff was attached to the right arm until a successful test was confirmed.

Whenever the result was outside the expected normal age related range, according to the National Heart, Lung and Blood Institute (NIH), USA, up to three new tests were conducted [25]. With results still outside normal range, a re-test was performed at a later occasion. If the values were persistently outside the normal range the values were reported to the HOPP team physician. The physician then contacted the parents and advised them to contact the child’s general practitioner for further follow-up.

##### 6) Blood samples

Blood samples are to be taken annually. However, not all children comply with this procedure. The children were given an opportunity to opt out on the blood sample test, while participating in the other test procedures. Hence, 58.4% (*n* = 1344) of the children completed blood sample procedures in a non-fasting state between 08.00 am and 13.30 pm at baseline. A similar number are expected in 2016.

Groups of four pupils were summoned from the classroom, making sure they had not performed strenuous exercise prior to sample collection. A phlebotomist collected samples in 4 mL tubes (Vacuette® Z Serum Sep Clot Activator and K2E K2EDTA) from the antecubital vein. The gel tubes were end turned and set to 30 min of coagulation before centrifuged at 2000 G for 10 min. Samples were transported to the laboratory and analyzed according to standard procedures. The executive laboratory at Vestfold Hospital Trust is certified according to NS-EN ISO 15189.

Samples were analysed for constituents related to physical capacity, dietary status, cardiovascular risk and inflammation. Ferritin, cobalamin (total vitamin B12) and holotranscobalamin (active vitamin B12) were measured on Architect i2000SR (Abbott Diagnostics, USA) with reagents from the supplier. Total cholesterol, HDL-cholestrol, iron and creatinine were measured on Vitros 5600 or Vitros 5.1 (Ortho-Clinical Diagnostics, USA) with reagents from the supplier. Micro C-reactive protein (mCRP) was measured on Vitros 5600 with reagents from Abbott Diagnostics. Blood counts were measured on Sysmex XE 2100 or XE 5000 (Sysmex, Europe) with reagents from the supplier. Hemoglobin A (HbA1c) test was measured on Tosoh G8 (Tosoh, Japan) with reagents from the supplier.

Blood samples were not taken fasting; hence triglycerides were not sampled. We were therefore not able to use Friedmans formula for estimating Low-density Lipoprotein Cholesterol (LDL-C). As a substitute non HDL-C were used (Serum cholesterol – HDL = non HDL-C) [26].

Any result outside the normal range, according to the laboratory at Vestfold Hospital Trust reference material used in the outpatient clinic, was reported to the team physician. He again contacted the parents to give appropriate advice, usually a follow-up at the child’s general practitioner.

##### 7) Quality of life (ILC)

A Norwegian version of the generic 7-item Inventory of Life Quality in Children and Adolescents (ILC) was used to assess various quality of life (QoL) parameters over the past week [27, 28]. The ILC includes a global QoL and scores seven items; school performance, family relations, peer relations, autonomy in play, physical health, mental health and a global assessment of wellbeing [29]. The children’s version of the ILC was completed on a hard copy form, under supervision. The test supervisors were trained in giving instructions for each question according to the ILC-manual.

##### 8) Oxygen uptake

Only pupils born 2008 are to be tested for oxygen uptake annually until 2021 as oxygen uptake procedures require use of highly sophisticated instruments for field-testing and is time consuming. By obtaining seven consecutive years of data, longitudinal measurement of physiological development across childhood will be available. In addition, the design permits comparison between intervention (*n* = 248) and control (*n* = 103) schools for all years.

The oxygen uptake test at baseline was performed on a treadmill (Matrix T3xe, USA) measuring peak oxygen uptake (VO_2peak_), respiratory rate (RR), respiratory exchange ratio (RER), peak ventilation (VE_peak_), peak heart rate (HF_peak_) and time to exhaustion using a portable metabolic analyzer, Cosmed K4b^2^,(Cosmed S.r.l., Roma, Italia). A Hans Rudolph V2 oro-nasal facemask (Hans-Rudolph, Shawnee, KS, USA) was used during testing. The protocol in use had a start load of 5 km/h – 5% incline for 5 min. The speed was increased to 7 km/h, and further by 1 km/h every minute until reaching 10 km/h. After this point, a 1% increase in incline was applied every minute until fatigue [30, 31]. Breath by breath results was averaged to 30 s intervals and the highest 30 s value near the end of the test was considered the VO_2peak_. As test termination criteria for adults are difficult to achieve for children, the children’s unwillingness to continue was used as an indication of VO_2peak_ [32].

### Tests not taken annually

Due to time restraints, suitability and validation some tests were conducted only at specific ages. Eriksen Flanker test for executive functions is validated up to 3rd grade. Older pupils used the Stroop test. Also, the Strength and Difficulties Questionnaire (SDQ), Self-Perception Profile for Adolescents (SPPA), the nutrition test and academic performance, were all used in specific age groups. Effects of the intervention will be investigated in regards to these test outcomes as well as secular trends over time. Also, the results will be compared to reference materials.

#### Eriksen flanker test

**Fig. 4:**
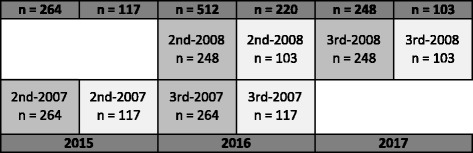
The expected number of participants, assuming no dropouts, performing Eriksen Flanker test each year is shown. The grade and birth year are displayed within each column along with the estimated number of participants. Dark grey columns are intervention schools, light grey control schools

The Eriksen Fish Flanker test (Millisecond Software, Seattle, Washington, USA) was used to assess executive function at specific ages (Fig. 4) [33]. A total of 120 experimental trials, 60 compatible and 60 incompatible trials, were completed after practice. The trial types appeared randomly and were equally likely to occur. The children had one minute break after every 40 trials [34]. The outcomes measures are reaction time and accuracy (percentage of possible trials where the correct response was selected). Calculation of interference scores (incongruent – congruent) for both reaction time (RT) and accuracy will allow for interpretation of the flanker effect as the difference in speed and accuracy of information processing [35].

#### Stroop test

**Fig. 5:**
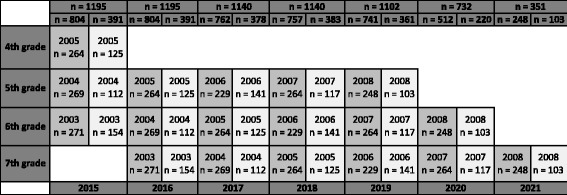
The expected number of participants, assuming no dropouts, performing the Stroop test each year is shown. The grade and birth year are displayed within each column along with the estimated number of participants. Dark grey columns are intervention schools, light grey control schools

The Stroop effect is the interference in the reaction time when the name of a colour is printed in a colour not denoted by the name (e.g., “red” printed in blue) [36]. The reaction time is faster when the name of the colour matches the ink colour (congruent) than when the name of the colour does not match the ink colour (incongruent) [37]. A computer-based method was used (Millisecond Software, Seattle, Washington, USA). The descriptions of the key-corresponding colour were in sight on the screen during the test. The pupils were told to respond correctly and quickly, using both hands. Reaction time and accuracy (percentage of possible trials with correct response) is the outcome. Calculation of interference scores (incongruent – congruent) for both reaction time and accuracy will allow for interpretation of the Stroop effect as the difference in speed and accuracy of information processing during the congruent and the incongruent trials [35]. Only certain grade levels, from 4th grade, will attend the Stroop test during the different test years (Fig. 5).

#### National academic tests

The Norwegian Directorate for Education and Training conducts compulsory national academic tests annually for 5th, 8th and 9th grades pupils. In national level of 5th grade reading, mathematics and English are published with mean values in a 50-point scale, with a SD on 10 points [38].

**Fig. 6:**
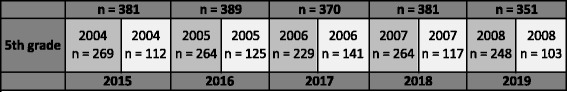
The expected number of pupils, assuming no dropouts, accumulating national academic tests until all pupils passed 5^th^ grade in the study period. Dark grey columns are intervention schools, light grey control schools. The year in each column corresponds to the birth year of the pupils

In the present study results from national tests for 5th grade pupils are collected (Fig. 6). The tests are performed in the fall each year; hence baseline data for the 5th^-2004^ grade pupils in 2015 was collected in fall 2014. As the study progress results from academic test will accumulate for 5th graders. In fall 2019, the last 5th grade (born 2008) will complete their national tests, hence secular trends will be reported. This also permits extraction of individual information about the academic achievement of each pupil at both intervention and control schools. In addition, comparison with national results is possible, however, no measure of one-year intervention effect is possible. [38].

#### Nutrition

The Norwegian developed and nationally validated dietary questionnaire “Ungkost 2000” concerning nutrition was completed using Questback [39, 40]. “Ungkost 2000” has 18 items including age, gender, PA level, inactivity and parents’ education level, diet and body weight [40]. The diet part of the questionnaire includes items as “ordinary Norwegian diet”, vegetarian/vegan, diabetes, allergies, as well as questions about weight satisfaction and attempts of weight loss. In addition, questions about “your diets role for your health” and if the child consider his/hers diet to be healthy or not are included. The questionnaire also comprises the number of times a week they eat breakfast, lunch, dinner and supper, in addition to how often they eat lunch during weekdays.

An extensive mapping of the amount of milk, orange juice and soft drinks (with and without sugar) is incorporated in the enquiry, as well as a variety of different food products including vegetables, bread, dairy products, fish, fast-food, sweets and vitamins. The enquiry gives a general description of different aspect of children’s nutrition status.

**Fig. 7:**
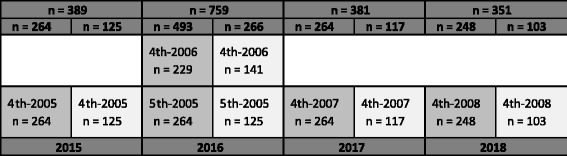
In 2015 the cohort of 4^th^-2005 grades perform the “Ungkost 2000” at baseline, and repeat the test as 5^th^ grade in 2016 as to measure effect of one year intervention. Each year a new cohort reach 4th grade and complete the test. Each cohort is marked with grade and year of birth, in addition expected estimated sample size, assuming no dropouts. Dark grey columns are intervention schools, light grey control schools

The “Ungkost 2000” is designed for 4^th^ grade pupils, and hence was given to 4th^-2005^ grades in 2015 (Fig. 7). To test the effect of one-year intervention “Ungkost 2000” is repeated for the same pupils in 2016 (5^th-2005^). Each year a new cohort reach 4^th^ grade and complete the test to accumulate data for measuring secular trends.

#### SDQ, SPPA and friendship nomination

The Strengths and Difficulties Questionnaire (SDQ), The Self-Perception Profile for Adolescents (SPPA) and friendship nomination were given digitally under supervision to pupils in 6^th^ and 7^th^ grades, using Questback. SDQ is a behavioural screening questionnaire for 2–17 year olds inquiring 25 positive and negative attributes [41]. SDQ is divided in five scales, each with five items; emotional symptoms, conduct problems, hyperactivity/inattention, peer relationship problems and pro-social behaviour. The first four items are added together to generate a total difficulties score. SDQ reflects levels of internalizing and externalizing psychopathology within a Norwegian child population [41–44].

SPPA has been widely used in research related to self-concept [20]. The revised version of SPPA was used in the Young in Norway study [45]. The SPPA consist of five items, each rated on a Likert-scale.

Children’s best friend nominations are thought to represent the peers with whom participants affiliate the most strongly [46]. Having close friends during childhood has been linked to improved mental health and resilience in later life [46]. Friendship will be evaluated by use of «best friend» nomination, including the listing of current friends and three best friends [47]. Also, the children will be asked to justify the reason for someone being their best friend. These answers will be organized into different categories as suggested by Thorpe et al. [48].

### Parental questionnaires

Parents annually received the parental version of ILC, which certifies for analyses of cross-sectional data at baseline as well as individual changes in the parental understanding of their child’s QoL across years [27, 28]. Annually an increasing number of tests will be accumulated for each grade as the study progresses, and secular trends for each age group will be established.

Parents also reported their children’s leisure time activity, any illnesses and parent’s education level. The leisure time questionnaires included three alternatives for non-physical activities, including type of activity, times per week and total hours a week. Similar question were given regarding PA, as well as any illness. Parental education level was divided in to elementary school, high school, bachelor degree and master/PhD degree. The parental questionnaire for children’s leisure activity was not validated and serves only as a report of leisure time activity.

#### Analysing tools

All data recorded on paper in the baseline tests were digitalized. The digitalized data from questionnaires were manually controlled for outliers and extreme values to improve validity. Ultimately, all data were unidentified and transferred to SPSS 21 (SPSS inc. Chicago, Illinois, USA) and other statistical software for analyses.

### Analytic possibilities

A longitudinal controlled intervention design like HOPP allows for multiple analyses, from cross-sectional analyse at baseline values, evaluation of effect after one-year intervention, longitudinal effects and secular trends.

#### Main outcome measure

In the present study cardio-metabolic risk factors were calculated by integrating objective measurements of BMI, waist circumference, muscle mass, percentage fat, endurance test, total serum cholesterol, HDL, non-HDL, mCRP and HbA1c. Firstly, all variables were divided into quartiles. Secondly, the results were dictomised as the children in the quartile of highest risk factor were given the score 1, and the children in the other quartiles were scored 0. Thirdly, children categorise with risk factors were assigned to one of 11 risk factor categories, 0–10, depending on their number of risk factors. Fourthly, the probability of children having 0 to 10 risk factors were calculated using the binominal probability formula (*n*! • *p* ^r^ • (1 – *p*)^*n-r*^ / (*r*! • (*n*-*r*)!) [49]. Where *n* is the possible number of risk factors (total of 11), *p* is the probability of having a risk factor (highest risk factor quartile: 0.25) and *r* is the number of risk factors for which the probability is calculated (0–10) [17]. Fifthly, the observed number of children in each group was compared to the expected number by calculating the ratio and plotting with a 95% confidence interval. Finally, a composite cardio-metabolic risk factor profile was calculated using Z-scores by age and gender for selected risk factors. The Z-scores were then summed for each individual regarding the individual risk factors to create the clustered risk score [17].

The occurrence of cardio-metabolic risk factors in the HOPP study was calculated at baseline. In addition, the calculus is to be computed annually to reveal any change in cardio-metabolic risk factors as a result of the intervention.

#### Baseline analyses

Baseline tests allows for cross sectional analyses for all variables. The large sample permits establishing representative normal materials for selected variables for each age group and gender. Inference between related variables may disclose health related correlations. Comparison with existing representative normal materials may document any changes regarding health related issues, for example an increase or decrease of overweight in the Norwegian child population.

Based on the cross-sectional data generated at baseline, the large sample size permits establishing normal materials in a variety of variables. In addition, multiple regression models were used to analyse inference between variables.

#### Analyses of effect

Establishing a baseline in 2015 permits a paired comparison of results in 2016, meaning one-year effect analyses of intervention on multiple variables as described in Table 2. The analyses would include test *within-subject* variation as well as *between-subject* variation for both intervention and control schools using basic univariate parametric tests as e.g. t-tests, linear or logistic regression analysis and linear mixed models for repeated measurements. Possible confounding, mediation and/or effect modification will be assessed using stratified and multivariable statistical models and statistical methods for causal inference (e.g. Directed Acyclic Graphs).

##### Intervention analyses

Annually analyses between intervention and control schools in numerous variables, the sample size for a parallel group design is calculated using *n* = 2 • (σ / Δ) ^2^ • *k* [26]. Here n represents the number of participants in each group, σ (sigma) is the expected standard deviation of the observations (which are assumed equal for all groups), Δ (delta) gives us the relevant difference between observations, and k is the constant in this study chosen based on the common values by a double-sided t-test set at 5% and with test strength 80% (this corresponds to a *k* of 7.9) [26]. Based on these calculations we have determined the sample size to be adequate for all variables for all ages, also when divided into gender (Table 3).

#### Analyses of longitudinal effect

The longitudinal intervention design allows for exploratory analysis of group means over time to discover patterns of systematic variation across groups, as well as among individuals. The design allows for analyses of age and growth effects [50]. As the eldest pupils enrolled at baseline 2015 finish elementary school in 2016, only one year of follow-up is possible. However, an increasing number of years of follow-up are possible with decreasing age at enrolment. This allows for an adjustment of the longevity of being enrolled in the program, and hence measurement of the time-in-the-program effect. Secondly, an annual comparison between the intervention schools and the control schools allows for testing of effect of the intervention. Thirdly, mean values across test time allows for time slopes showing possible effect as an interaction (i.e. effect modification) of intervention and time. A different approach for test across test time may be pre-post analysis of a single follow-up response in conjunction with a baseline measurement [50]. Fourthly, an annual comparison with the upcoming grade within the intervention schools allows for analyses of secular trends.

The longitudinal design in the HOPP study with repeated measures allows for use of a linear mixed regression model using test-year, years of follow-up, intervention versus control school, age and gender as fixed factors for the main outcome of cardio-metabolic risk factors. A similar model may be used for secondary outcomes of academic performance, PA level, cognitive performance, as well as medical and physical results, and simultaneously controlling for baseline values. In addition, the design admits analyses of interaction (i.e. effect modification) between type of intervention group and number of years in intervention.

#### Ethical considerations

Procedures and methods used correspond to the ethical guidelines defined by the World Medical Association’s Declaration of Helsinki and subsequent revisions [51]. The Regional Committee for Medical Research Ethics approved the study protocol (2014/2064/REK south-east) and the study is registered in Clinical trials (ClinicalTrials.gov Identifier: NCT02495714). Informed consent was obtained from all parents prior to testing. No identifiable data is to be published.

The HOPP study is exposed to three challenges regarding the sample size; 1) unexpected dropouts of pupils across years, 2) calculated dropouts of pupils to junior high school, and 3) drop-ins as the control schools may introduce similar interventions. However, the control schools do currently not have any PA program or plans for establishing any such program.

## Discussion

In the western world with increasing inactivity and obesity increasing PA in children is a goal in itself. To counteract health related problems in childhood, school-based PA programs may become an important tool [8, 9, 52, 53]. There are studies showing that an increase in PA may simultaneously improve academic performance even without a pedagogical approach included in the activity [54]. A strategy combining academic learning with increased PA may subsequently improve both academic performance as well as physical health in the childhood population [8, 9, 52, 53]. The ability to reach all socioeconomic layers of the population is a great strength of school-based PA programs as it may contribute to reduce social inequalities in health.

Waters et al. demonstrated that child obesity prevention programmes were effective at reducing adiposity, although not all individual interventions were effective [10]. A recent review by Mura et al. assessing 47 school based RCT ‘s on PA interventions also showed promising positive results for cardio-metabolic variables and physical fitness [7]. A longitudinal study permits the intervention to work over time and may create habitual processes in the pupil’s everyday school life.

Lately two Cochrane reports included several studies focusing on health results from schools based RCT’s [9, 52]. Primary outcomes were rate of moderate to vigorous PA (MVPA) during the school day, time in MVPA during school day and time spent in front of a screen. Secondary endpoints were blood pressure, cholesterol levels, BMI, peak oxygen uptake and heart rate [9]. The reports displayed positive health effects, hence school based interventions were recommended [52]. However, as forty-four studies were ranked in four quality levels, none were ranged above “low quality” [9]. The 2013 report showed a positive effect in more time spent in MVPA, less time in front of a screen, higher VO_2max_ and reduction in serum cholesterol values. The effect sizes were small on all outcomes, and Dobbins et al. warn that despite the inclusion of several RCT’s with a large number of participants, a moderate risk of bias is present and any interpretation should be done with caution [9]. Both reports concluded that additional research on the long-term effect is warranted [9, 52]. The longitudinal design in the HOPP-study addresses this issue by allowing follow-up of 6 years for the youngest children.

Most school based PA intervention studies focus on risk factors of cardio-metabolic disease and academic performance, however, few studies have included measurements of psychological and cognitive outcomes [7]. A review study by Taras et al. found short-term effect on academic results after intervention of increased PA, but uncertainty regarding the long-term effects [54]. Singh et al. found a positive link between participation in PA and long-term effects on academic results [8]. Martin et al. investigated lifestyle changes for overweight children and its effect on academic results in a meta-analysis [53]. The conclusion was despite the lack of clear results, many children profit academically from school based lifestyle interventions. However, better-designed studies are needed to investigate the association between academic, cognitive and physical outcomes with overweight and obese children [53]. The longitudinal design in HOPP permits investigation of long-term associations between PA and cognitive and academic results. The HOPP intervention with its increased PA level is believed to have a significant impact on the children’s cognitive skills. The use of standardized cognitive and academic tests in association with objective assessment of PA level admits valid evaluation of the outcome.

Children, who spend one third of their lives at school, develop significantly more traits than physical lifestyle and academic skills during their school years. Therefore, it is surprisingly few studies who include measurements of emotions, nutrition intake and quality of life [7]. Studies do show effects of exercise beyond improvements in physical fitness and body composition, as in mental wellbeing, psychosocial outcomes and behavior [55]. The present study acknowledges this lack of information and has included standardized tests mapping quality of life (ILC) for all participating children. In addition, age-specific tests of mental health (SDQ) as well as questionnaire concerning body image (SPPA).

Earlier studies have suffered from limited number of participants and study length. Most studies in the review of Singh et al. [8] had a cross-sectional design [56–65], few were interventional studies [18, 66–68], and a only two had a longitudinal design [18, 59]. Both Lees et al. and Mura et al. conclude that larger school-based PA interventions are needed and should focus on cognitive abilities, psychosocial functioning and behavior as well as cardio-metabolic risk factors [7, 55]. The overall conclusion is that several smaller studies display a positive effect of school-based PA programs, however, few large longitudinal studies have been completed to demonstrate similar findings. HOPP, with its longitudinal design and large sample size across six grades, has an ability to evaluate the effect of school-based PA program over time, mainly on cardio-metabolic risk factors, but also in several other variables. The results may therefore yield necessary knowledge to provide a foundation for policy making and future guidelines regarding PA in school. Implementation of health promoting behaviour in elementary schools, with the prospect of reaching all socio-economic layers in a child population, may have the possibility of preventing lifestyle related diseases. Doing so, school-based PA program in elementary schools may have the potential to even out socioeconomic inequalities in public health. The benefits may potentially be large as these diseases are regarded as a major concern against public health [9, 52, 53].

The society may experience severe socioeconomically consequences if non-commensurable diseases increase further, as the cost of treating these diseases vastly exceeds the cost of preventing them. Implementing a school-based PA program as lifestyle adjusting measure is not expensive. A comprehensive schooling of teachers beyond prerequisite education in school-based PA program is required, as well as establishing a set of teaching material. However, these measures are only implemented once and therefore have limited economical consequence. With its longitudinal design, HOPP may be able to disclose effective strategies to reduce cardio-metabolic risk factors in a child population in an affordable way. If positive findings are revealed as an effect of HOPP, a national implementation of increased daily PA in elementary schools may have an profound effect on current and future public health.

### Limitations of the study

One challenge with all longitudinal designs, is dropout as the participants decline completing the intervention or do not show to tests. In addition to regular dropouts, the HOPP study has a decreasing number of participants as the children are transferred to junior high school. This design have two major implications: ^1)^ the amount of years the pupils are in the intervention varies depending on the age at inclusion. Children born in 2003 only participate one year before exit. Those born 2004 are enrolled in two years, born 2005 in three years, and so forth. This implies challenges regarding the statistical analyses, as results from pupils with fewer years in intervention must be weighted less than pupils with additional years. ^2)^ Due to age-specific tests not all children receive all tests. In those tests only a limited amount of participants are to be analysed in an effect of intervention. Instead secular trends are to be used.

A major challenge in all interventions is compliance, and in the present study the teacher’s compliance is essential. Encouraging teacher’s to complete the intervention every day is a major challenge, and is taken care of by Horten municipality. Two designated teachers at each school as well as a centralised coordinator, performed daily encouragements as well as written control of participation. The individual compliance of each pupil is more difficult to survey. The participation rate as being part of the class is registered using reports of absence from school. However, in what extent and what intensity each pupil performed each task are more difficult to control.

Due to Horten municipality decision on giving all schools in the municipality the same school-based PA program, a randomised controlled trial was not possible. However, using control schools with corresponding socioeconomic status, a controlled design was used.

## Conclusions

The HOPP study will give valuable information about a broad spectrum of outcomes following a low-cost PA intervention. A positive finding in health factors using PA during school hours, simultaneously with enhanced effect of academic performance may positively affect politicians’ and teachers’ view of how the pedagogical approach to learning could be organized. Positive results from HOPP may impact the scientific and political arena to recognize the importance of implementing school-based PA program as a part of reaching both educational and public health targets.

## Abbreviations

HbA1c: Haemoglobin A1c is glycated haemoglobin (three-months average plasma glucose concentration
HDL: High Density Lipoprotein
HF_peak_: Peak heart rate
HOPP: The Health Oriented Pedagogical Project
ILC: Life Quality in Children and Adolescents
isoBMI: Iso Body Mass Index
mCRP: micro C-reactive protein
MVPA: Moderate to Vigorous PA
NIH: National Heart, Lung and Blood Institute
Non HDL-C: Serum cholesterol - High Density Lipoprotein
PA: Physical activity
RCT: Randomised Controlled Trial
RER: Respiratory Exchange Ratio
RR: Respiratory rate
SDQ: Strength and Difficulties questionnaire
SPPA: Self-Perception Profile for Adolescents
SPSS: Statistical Package for Social Science
VE_peak_: Peak ventilation
VO_2peak_: Peak oxygen uptake
